# Changes and Forms of Sexual Behaviour in old age: A Qualitative Study of Older People in Southeastern Nigeria

**DOI:** 10.1007/s12119-023-10076-0

**Published:** 2023-03-30

**Authors:** Stephen Sunday Ede, Gloria Chepngeno-Langat, Chisom Favour Okoh

**Affiliations:** 1 Department of Gerontology, Faculty of Social Sciences, University of Southampton, Southampton, UK; 2 Department of Physiotherapy, Faculty of Allied and Health Sciences, Gregory University, Uturu, Nigeria; 3 School of Sports and Health Sciences, University of Central Lancashire, Central Lancashire, UK; 4 African Population and Health Research Centre, Aging and Development Unit, Nairobi, Kenya

**Keywords:** Intimate sexual behaviour, Nigeria, Older people, Physical sexual behaviour, Sexuality, Sexual desire

## Abstract

The misconceptions that old age is an asexual phase of human life has been challenged by increasing empirical evidence which shows that sexual activity persists in old age albeit in different forms and frequency. This study examined how a group of older people in southeastern Nigeria express their sexual behaviour. The semi-structured individual interviews with 14 older people (9 male, 5 female) aged 60?89 years were conducted using an exploratory qualitative approach. The data generated were analysed thematically, and two themes were conceptualised including *diverse sexual behaviour* and *mutual understanding*. These themes supported a pattern among the participants where there is a drop in the frequency of physical sexual behaviour but their sexual interests were described to be more stable. However, the sexual interest is channelled into more intimate sexual behaviour. Thus, sexual behaviours in later life in this study were not declining but showed diversity and modifications; most have adjusted to incorporate more emotional bonding and caring. More so, what forms of sexual behaviour constitute for these older partners are often linked to the presence of dynamic interplay of influencing factors ingrained on the level of the older partners mutuality in understanding and responding to theencroaching age-related changes in their sexual behaviour. Remarkably, these factors could be controlled, which signposts a potential premise and point of policy and practical intervention to promote healthy sexual behaviour in later life.

## Introduction

A typical conflicting subject in older people’s sexual behaviour is distinguishing the thin line between age-related declines in the frequency of penetrative sex and the notion that old age is an asexual phase of human life (Nyanzi, 2011; Agunbiade & Gilbert, 2020). It is not until recently that research and policy have identified sexuality as a right for people of all ages and sex (Chepngeno-Langat & Hosegood, 2017), and emerging studies have demonstrated that the sexuality in later life is connected to healthy ageing as it has been shown to influence their quality of life and perceived life satisfaction (Hardy et al., 2010; Jeste et al., 2013).

Lindau et al. (2007) define sexual behaviour as “any mutually voluntary activity with another person that involves sexual bonding, whether or not intercourse or orgasm occurs” (Lindau et al., 2007:763). Hence, sexual behaviour could be vaginal intercourse or other alternatives such as oral sex, touching and caressing, masturbation, companionship, and the individual meaning attributed to sexual relationships (Clarke, 2006; Da Silva, Pelzer, & Da Silva, 2019; Agunbiade & Gilbert, 2020). This study aligns with this definition of sexual behaviour as having two-component: physical sexual behaviour or sexual activities, and intimate sexual behaviour such as bonding, companionship, and caring. This study also aligns with previous studies (Chepngeno-Langat & Hosegood, 2017; Traeen et al., 2018), which proposed that although there may be changes in some older people’s sexual behaviour due to age-related factors, older people might be able to adjust and adopt behaviours through which their sexual satisfaction is maintained. Age-related developments such as frailty, changes in living arrangement, and loss of partner, do not always lead to a decrease in sexual desire; men and women do have sexual urges and remain sexually active even at advanced age (DeLamater, 2012; Freeman & Coast, 2014).

Presently, most data on sexual behaviour have focused its discourse around vaginal intercourse, which is partly due to initial medicalized focus, and due to the conservative societies like Ayalon et al. (2019) reported that older Israeli interviewees rarely mentions other alternative sexual behaviour in their interview research among 24 men and 23 women aged 60 and older. This study confirmed the findings of quantitative studies about the conservatism and how it negatively impacts sexual behaviour in later life. However, an emerging body of literature on sexual behaviour suggests that older adults are engaging in different forms of sexual activity. For example, Karraker & DeLamater (2013) discovered that 90% of married older people (aged 57 to 85) in USA reported having regular foreplay, and 10% had oral sex in the previous year. In another survey conducted in Spain, Palacios-Ceña et al. (2012) discovered that among older people aged 65–74 years, 82% of men and 73% of women mentioned kissing and hugging in the previous year, and 11% and 5% reported oral sex. However, most of these studies were conducted in developed or middle-income countries with the participants drawn largely from European, with a crucial gap in its generalizability to sub-Saharan African.

Given that no right narrative can effectively explain all older people in terms of sexual activity or interest, more research across cultures is needed to emphasize generally observed patterns and causes of changes in sexual activity.

The aim of this study is to understand older people’s interpretations and expressions of sexuality as they adapt their sexual behaviour in light of age-related changes to ensure that their sexual pleasure and satisfaction is maintained. This study is delimited to sexual behavioural aspect of human sexuality within heterosexual relationships among older people in Southeastern Nigeria to answer the research question of ‘what changes and forms of sexual behaviour exist in old age? Understanding what constitute changes and forms of sexual behaviour for older people will aid in highlighting their influencing factors, thereby contributing to promoting healthy and successful ageing in this population.

### Sexual Desire and Sexual Activity in Older age

Sexual desire acts as a motivational force and prerequisite for individuals’ sexual behaviour (DeLamater, 2012; Fischer et al., 2020). Evidence in the literature for changing sexual desire with age have reported varied patterns across culture. For instance, Da Silva et al. (2019) conducted a descriptive study using a qualitative approach to explore Brazilians older women’s sexuality. Some of their interviewees’ response demonstrated that though age comes with some limitation, it does not prevent them from relating sexually as the interest and natural urge do not extinguish. In four European countries, marital duration and being a woman were reported to be associated with more decline in sexual desire of people aged 60–75-year (Carvalheira et al., 2019). In a qualitative study by Delamater, Koepsel, & Johnson (2019) among 24 heterosexual and Caucasian women aged 55–80 in the USA who were interviewed about their recent sexual behaviours, most of the women except three reported a decline in sexual desire which they attributed to health factors, menopause, and presence of a partner, as well as perception and attitudes of the older person.

Similarly to sexual desires, there are mixed findings in the literature regarding the pattern and changes in older people’s sexual activities. For instance, Ayalon et al. (2019); Ricoy-Cano, Obrero-Gaitán, & Caravaca-Sánchez, (2020) reported that there is a much decline in sexual activity in older age in their semi-structured study among Israeli and systematic review respectively. Whereas, Delamater et al. (2019) in their study, stating that some older people have stable sexual activity over time whereas others become more sexually active in later years, as older age features like retirement could provide them with more time and freedom to engage in sexual activity spontaneously. Freixas, Luque, & Reina (2015) and Lindau & Gavrilova (2010) also noted an increase in sexual activities with age among older women in Spain and older adults in USA respectively.

### Intimate Sexual Behaviour

Emerging studies have begun to identify the intimate component of later life sexual behaviour as a response to the critics of previously medicalized studies (Traeen et al., 2018; Fischer et al., 2020). For instance, GewirtzMeydan & Ayalon (2020) online survey to understand public opinion found that older people’s sexuality was regarded as decreasing, conservative, and uninteresting, but it was also described as containing more emotions and sentiments; being better and more complete. Although this survey had more focus on physical sexual behaviour, it identified the importance of intimate components of later life sexual behaviour. Similarly, a survey by Štulhofer et al. (2020) identified from a cross-cultural (n = 270 Norway, 1045 Denmark, 990 Belgium, 509 Portugal) sample of older people aged 60–75 years that emotional intimacy was associated with frequency of sexual intercourse and contributes to sexual well-being in later life.

Additionally, von Humboldt et al. (2021) qualitative interviews with 495 older adults (N = 168 Portuguese, 171 English, and 156 Brazilian) aged 65–98 years sought to investigate how older people express themselves sexually across different cultures. They highlighted the presence of some intimate sexual behaviour in later life including tenderness and care, altruism and gratitude, positive communication, and supportive relationship. Their work makes a landmark contribution to this topic with a major focus on intimate sexual behaviours. Although its interview discourse was narrow, using only two interview questions centred on ‘current sexual expression and things important for their sexual expression’.

### This Present Study

The reviewed literature demonstrate a shift from a medicalized and negative social construction of older age as an asexual state, towards a better awareness on the importance of sexual behaviours in older age to their overall well-being (WHO, 2015; Fischer et al., 2021), cardiovascular health, increases relaxation and reducing pain (Syme et al., 2013). Thus, this present study aligned with the argument that physical changes associated with advancing age in some older people do not necessarily reduce the opportunity to explore their sexual behaviour. Instead, alternative interpretations and expressions of sexuality may be one of the great opportunities of growing older. As recommended by Bell et al. (2017), this study will concentrate on the changes in sexual desire, motivation, and behaviour, as well as the factors that influence these changes. The present study seeks to ascertain whether conservatism in some African cultures compared to most Western societies (Fischer et al., 2021) and the cultural dynamics of sexual behaviour (Khumalo et al., 2020), could present differing patterns and sexual activities. Palacios-Ceña et al. (2012) noted that alternative sexual behaviours such as kissing, oral sex, and masturbation may be missing or suppressed by societal norms in conservative cultures, making adapting to sexual behaviour in later life more challenging or nonexistent. Agunbiade and his colleagues have done three related works among Yoruba ethnic group in the western part of Nigeria (Agunbiade, 2013; Agunbiade & Ayotunde, 2012; Agunbiade & Gilbert, 2020). However, these qualitative works often adopted the medicalized view of sexual behaviour around penetrative sex only. Therefore, this study seeks to add to the literature on older people’s interpretations and expressions of sexuality in south eastern Nigeria.

## Study Setting and Methods

### Study Setting

This study was conducted in Southeastern region of Nigeria, home to the Igbo ethnic group, with a population of around 8 million according to the 2006 census and with one of the highest population density in Africa (Okafor, Adeleke, & Opara, 2007). The predominant religion is Christianity (90%) (Offor, 2020) but traditional cultural heritage still has strong adherence and presence especially among older people (Iheanacho, 2009; Hale, 2006).

The Igbos traditional socio-cultural sexuality setting is characterized by polygamy and patriarchal family system (Uchendu, 2007), with one-third of married women in polygamous unions (Arthi & Fenske, 2018), and conservative social norms (Agunbiade & Ayotunde, 2012) where cultural scripts for sexuality is heavily gendered. However, contemporary Igbo society have continued to witness a massive conversion in values and a strong Christian influence for instance propagating monogamy as the legitimate family system (Okeke et al., 2017). Thus, more researches are needed to update the nature of sexuality in later life.

### Research Design

A qualitative research method was used involving face-to-face semi-structured interviews. A purposive sampling technique was used to recruit participants 60 years or older which ensured data was collected from a diverse population (Ormston et al., 2014; Mason, 2002). Characteristics such as age range, sex, occupation, education, religion, family system, and resident area were used as shown in Table 1. Only married people or those with a long-term sexual partner were eligible for this study to allow for sensitivity since, in the religious and socio-cultural settings of southeastern Nigeria, sexual behaviours is only approved in marriage or a long-term partner relationship (Ojo, 2005). Furthermore, the questions were in reference to recent sexual behaviour with a current sexual partner. Thus, those widowed, unmarried, or divorced, were excluded. Participants were recruited through public institutions including pensioners’ centres, churches, and older peoples’ peer groups. Fourteen (9 male; 5 female) older people aged 60–89 years participated in this study.

**Table 1:** Demographic Characteristics of the Participants

Pseudonym	Age range	Sex	Occupation	Education	Religion	Family system	Resident area
Abara	70–79	M	Security personnel	Secondary	Christian	Monogamy	Suburban
Adaoma	60–69	F	Teacher	Secondary	Christian	Polygamy	Rural
Akama	70–79	M	Lawyer; pastor	MSc	Christian	Monogamy	Urban
Awoke	60–69	M	Farmer	Primary	Christian	Monogamy	Urban
Daberechi	60–69	M	Anglican priest	Bsc	Christian	Monogamy	Urban
Duruigbo	70–79	M	Driver	Primary	ATR	Polygamy	Rural
Papa	70–79	M	Tailor	Tertiary/theocratic school	Christian	Monogamy	Suburban
Chioma	80–89	F	Businesswoman	OND	ATR	Remarriage	Rural
Ngozika	60–69	F	Professional Cook	Secondary	Christian	Monogamy	Urban
Nwamajoka	70–79	M	Civil servant	Bsc	Christian	Monogamy	Suburban
Nwanga	70–79	M	Farmer	No formal education	ATR	Polygamy	Rural
Odogwu	70–79	M	Lecturer	PhD	Christian	Monogamy	Urban
Oluchi	80–89	F	Retired civil servant, Businesswoman	Bsc	Christian	Monogamy	Urban
Zubechi	60–69	F	Typists	OND	Christian	Monogamy	Suburban

Key: ATR-African traditional religion

The interviews were conducted in August 2021 in either of English or Igbo language and lasted between 30 and 90 min depending on the participants’ capacities to “interact, conceptualize and remember” (Mason, 2002: 64). The lead author (male) conducted the interview alone under the supervision and guidance of two supervisors. The central focus of the interview questions guide (provided in supplementary material I) bordered on participants’ demographic characteristics, their current sexual behaviour, sexual interest, motivation towards sex, and whether there are changes on sexual activity compared to their earlier ages.

The interview was recorded with an android phone and transcribed verbatim in the English language with the Igbo interviews translated directly into English while transcribing.

Ethics Approval for this study was granted by the University of Southampton Ethics and Research Governance Online II (ERGO II). The interviews were carried out keeping with the COVID-19 precautionary safety measures of physical distancing, wearing a mask, and staying in a well-ventilated space (WHO, 2020). Consent of participants recruited for the study was sought with verbal and signed informed consent.

### Data Analysis

According to Clarke & Braun (2013), Braun & Clarke (2006), the thematic analytical approach in qualitative analysis can be theoretically flexible as it permits the application of different theoretical frameworks in the analysis. Thus, the critical realist ontology and the interpretive epistemological position were adopted when analysing the data. A critical realist ontology enabled the data to be understood as a phenomenon created and recreated through their social interaction including the active influence of the researcher’s interpretation (Ormston et al., 2014; Bryman, 2016). Conversely, the interpretivist epistemological position permitted interpretation of the observed data of the participants in understanding sexual behaviour in later life (Ormston et al., 2014).

The reflexive thematic analytical approach was used to code the data (Clarke & Braun, 2019; 2021). The interview transcripts were imported into NVivo12 software, which was utilized to organize and ‘get a handle’ on the data (Mason, 2002: 171). An open coding approach was utilized where the emerging codes were used as shorthand devices to label, separate, and organize data (Bryman, 2016:578). Each code was subsequently interrogated using the guide proffered by Lofland & Lofland (1995) to assess what category, concept, topic, and /or question about a topic each item of data represented. Where necessary, memos were created to guide the reflection and help as a reminder about analytical ideas and what each item of data represents. The codes were further reviewed concerning the transcript, during which some codes were merged, some labels were changed or deleted, and a coding tree was formed by categorizing similar concepts together and grouping them under a higher-order conceptual category (Bryman, 2016).

In each phase in the coding process, the emerging codes were double-checked across all the transcripts iteratively to identify every instance where they are applicable and to maintain a close link with data and the emerging concepts (Strauss & Corbin, 1998). The analysis process and the findings were reviewed with the two supervisors and areas of disagreement or discrepancies were revisited according to their recommendation until clarity is established.

After the coding process was completed, each code was then reviewed by comparing the concept as perceived and reported across the participants (Bazeley, 2009). The codes were then integrated; including memos on recurring ideas and topics to build up themes from the reoccurring topics and ideas that established the significance of the codes to the phenomenon of changes in later life sexual behaviour (Ritchie et al., 2003; Braun & Clarke, 2006).

The themes were also reviewed using the matrix query functions in Nvivo12 to identify and compare contrasting categories where they exist in the data by continually interrogating the created theme to check if it suits the raw data (Johnson et al., 2020). The themes that were created from the data during the matrix query support an overarching theme of *diverse sexual behaviour* among the participants.

## Discussion of Findings

This qualitative research explored how older people in southeastern Nigeria interpret and express their sexual behaviour in later life. From the data analysis, an overarching theme of *diverse sexual behaviour* was created as an appropriate description of the reported later life changes in sexual behaviour among the participants. There also the theme of *mutual understanding among partners* of the older adult throughout their life course.

### Diverse Sexual Behaviour

Across the transcript, the interviewees identified and defined their sexual behaviour in the earlier ages as one characterized by fun, pleasurable emotional bonding, frequent sexual activities and heightened sexual interests.

*“…things that concerns husband and wife you know at the beginning is always full of pleasure, fun and looking up to it every day”* (Awoke, M, 60–69).

However, good level of diversity in behaviour was demonstrated as different pattern was reported in older age where some had a drop in the frequency of physical sexual activities, while sexual interests were described to be more stable. Instead, the sexual interest is channelled into more intimate sexual behaviour. This is expressed below in the response of a driver and an older woman in remarriage:

***“****Duruigbo*.

… *If you do work that take strength … your work will affect you when you come back. You will be tired if you want to do it because it is the same strength you are talking about.*

### Researcher

*So do you mean the interest is still the same only the strength is going down?*

### Duruigbo

*Yes, the strength is the problem”* (Duruigbo, M, 70–79).

*“…our sexual urge might be there but the sexual intercourse is now interpreted or channel to other parts of our behaviour”*(Chioma, F, 80–89).

This findings aligns with evidence in the literature that older people’s sexual behaviour is individualized, as there is no “right” narrative that effectively describes all older people’s sexual activities (Mitchell et al., 2013; Da Silva et al., 2019).

### Physical Sexual Behaviour

The changes in physical component of their sexual behaviour received the most mention, especially the penile-vagina sexual intercourse as opposed to intimate sexual behaviours. For instance, when asked ‘how would you describe your current sexual behaviour?’ without definition and further probe, the participants were often drawn to understand and define it from the perspective of sexual intercourse, probably because this is the form of sexual behaviour common to their everyday social discourse. Thus, in most interviews, the interviewees first report that their sexual activities has witnessed a drop throughout the life course.

*“As weather people say; the higher you go, the cooler it becomes. We still engage in sexual behaviour but has reduced like once a month”*(Duruigbo, M, 70–79).

Although the degree of these changes differed, for instance, while many of the interviewees explained that they have dropped the frequency from daily to monthly, and other variabilities, some described a change from multiple times to at least once and with a focus on quality:

*“…you go so many rounds at night like four times when you are younger but this time twice or you can at least do once and stroke and conversation and until you sleep…”*(Papa, M, 70–79).

*“…sometimes we will be lying down, cuddle ourselves, sometimes end up having sex but even at that, it will not be as hot as it used to be…”*(Oluchi, F, 80–89).

This finding is consistent with previous studies in the literature that have acknowledged a wide variability of changes in sexual activities (DeLamater, 2012; Ayalon et al., 2019; Da Silva et al., 2019). Meanwhile, this is a novel finding within sub-Saharan Africa as studies in this region (Freeman & Coast, 2014; Agunbiade & Ayotunde, 2012; Agunbiade & Gilbert, 2020) had only reported a decline in sexual activities without acknowledging the variability of these changes. Major reasons for this difference in their finding could be attributed to the medicalized approach in their measure of sexual activities where it focused solely on penetrative sex without covering other sexual activities.

Instead, in this study, the sexual behaviour changes from focus on frequency to a slower, more sustained approach, while also communicating physically and emotionally. In most cases, these changes were understood as normal for their ages. Meanwhile, there were some contrasting responses. Like those who reported no change in their sexual behaviour throughout the life course:

*“…to me, sex is a natural thing. In my own experience, …I can say it is still the same. There have been no change in my sexual behaviour”*(Odogwu, M, 70–79).

A further probe to understand the contrast was explained by some interviewees to be due to influencing factors that encouraged their sexual behaviour:

***“****…My wife has the same characteristics as I. she is a professional. …like, we do our normal physical activities and take good care of our body. …If there’s any change I have not observed”*(Odogwu, M, 70–79).

While such narratives as Odogwu is diverse to the general pattern, it points to the very crucial influencing factors of later life sexual behaviour, which produces individualized sexual behaviour depending on the interplay between these factors as detailed in the subsequent sections.

### Sexual Interests

While the participants were most likely to focus on physical sexual behaviours, the interview guide was structured to get the interviewee talking in-depth on the comprehensive state of their current sexual behaviour including the intimate component. Thus, those who reported no change in sexual interest when probed further mostly explained that they have channeled their urge into more intimate sexual behaviour.

*“…our sexual urge might be there but the sexual intercourse is now interpreted or channel to other parts of our behaviour”*(Chioma, F, 80–89).

Thus, while Freeman & Coast (2014) reported that, the continuous desire for sexual activities caused frustration on the older Malawians stemming from inability to maintain their usual sexual frequency, the present study finding contrarily support the findings that sexual desire commonly stood out as a more stable expression, which does not wane with age and longer marital duration, though one may engage in physical sexual activities less frequently (Arias-Castillo et al., 2009; DeLamater & Koepsel, 2015), and is suggestive that the participants often channelled their sexual desires into more intimate sexual behaviour (Yang et al., 2018). This finding agrees with the assumption of this present study and confirms the established evidence in the literature that the older age is not an asexual period (Nyanzi, 2011; Ricoy-Cano et al., 2020).

More so, there are some who explained that they have inherently low interest in sexual needs that have persisted from their younger age. Especially, people that experienced a conservative or repressive socialization and the female participants were more likely to report the low interest in sexual needs. For example, Oluchi’s response to the question on whether religious practices and teachings influenced her sexual behaviour in older age was:

*“…you are taught that premarital sex is a sin… when you have been learning how to hold yourself from a young age it will also enter into the old age. You have good self-control and sex is not something you are mad about”* (Oluchi, F, 80–89).

For Oluchi, her sexual behaviour is dependent on the partner’s request or for the sake of procreation. Otherwise, they have no personal interest. Besides, women were more likely to report low sexual interest, which is consistent with previous studies that men compared with women, are more likely to show more interest in sexual behaviour (Ayalon et al., 2019; Lindau & Gavrilova, 2010), which could be due to society expectations or stereotypes.

### Intimate Sexual Behaviour

The commonly mentioned intimate sexual behaviour includes companionship, deeper communication, and caring for each other, as well as love, and bonding. For instance, closeness times are used to look back in the marital journey, also to discuss the state of things in their homes.

*“…there is happiness that is shared within the family. …when I come back [from day activities] I tell her the deeds of the day, …these moments of reflection help to increase the bond I have with her. I also share ideas and plans with her…”*(Awoke, M, 60–69).

A further probe to understand how the intimate sexual behaviour in forms of love and caring has changed over the years showed that it is an aspect of the older people’s sexual behaviour where they have adapted and developed more in light of the age-related changes to ensure that their sexual pleasure and satisfaction are maintained, which was similarly suggested by Chepngeno-Langat & Hosegood (2017), and Traeen et al. (2018). Thus, similar to previous findings (Ayalon et al., 2019; Štulhofer et al., 2020), the present study suggest more shift towards intimate aspects of sexual behaviour as in companionship, deeper communication, and emotions and caring, as well as love and bonding. The women participants were most likely to talk about the importance of love and caring as the most required behaviour to sustain the remaining part of their marriage. The interviewees explained that though love is existing from a younger age, at the older age it gets more real, true, and it is most needed because the age-related changes in some could tend to reduce the physical beauty and pleasure that drive intimacy at younger age. Instead in later life, There seems a crucial shift in understandings of the body with ageing and about touch and intimacy in the context of physical change, decline and the experience of pain or ill-health.

*“Love and character… …the change will be there. …, you will still be seeing each other like at the beginning but not in terms of sex and kissing. Rather, what you will now be thinking of is…, how your body is, let me touch the part that is paining you…”*(Adaoma, F, 60–69).

“*Other ways of relating are to sit down and chat and discuss and laugh …sometimes, I massage his body …especially when he is not feeling too well?…*”(Oluchi, F, 80–89).

However, while the pattern in intimate sexual behaviour of the interviewees was commonly identified to be more frequent, there exist some contrasting reports from some participants who explained that they were not so much proud of their sexual behaviour. Their reasons often include the depriving effect of polygamy on the first wife that gets her more isolated and ignored in the older age. Additionally, there were those that accepted to have had more crisis and loss of trust over the years, like in the response of an older woman whose husband was frail when asked how their sexual behaviour is being maintained:

*“It is mainly maintained by a trust for me oh. …when both of us were younger, he trusted me so much. …because my husband is older and not very in for it, he hardly wants to notice any different movement without assuming maybe am seeing another person. That singular act has not been helpful… So I think trust is vital”*(Ngozika, F, 60–69).

Thus, there is an individualized or diverse physical and intimate sexual behaviour and there is no homogenous description for all the older people in this study. Instead, several events and experiences that the older people are exposed to over their life course and their prevailing sociocultural environment depicted a vital link to the sexual behaviour maintained in later life. This finding supports the gerontologists’ claim that ageing is a diverse process that is experienced very differently by different individuals (Franceschi et al., 2018). As well, it supports the intersectionality theory, which recognizes that when two or more factors interact to affect behaviour, a different effect is produced (Clarke, 2006; Calasanti & King, 2015).

This finding is novel among studies in sub-Saharan Africa and supports the recent studies in the literature (Traeen et al., 2018; Fischer et al., 2020; Gewirtz-Meydan & Ayalon, 2020) that have acknowledged and considered the intimate forms of sexual behaviour rather than the medicalized and physical perspective of sexual activities that initially dominate the literature. It also highlights a potential area of older people’s life that is linked with their healthy ageing as it touches the biopsychosocial aspect of their later life (Towler et al., 2022) as detailed in the subsequent sections.

The subsequent sections explore in-depth the interconnected factors reported to influence later life’s sexual behaviour.

### Mutual Understanding

Although older people in this study developed more intimate sexual behaviour over the years, these study findings also suggested that there is a diversity identifiable with each individual such that partners experience depend on interaction with their environment. Mutual understanding in this study is defined as the partners’ ability to see things from the lens of another and cooperate to adjust or adopt and explore more ways to express their sexual behaviour despite the factors present in their environment. The theme of mutual understanding explains what forms of sexual behaviour an individual older person could maintain depending on how the partners handle their age-related changes and emerging partner differences (especially, the intimate component). An example is in the response of those who reported having developed more in their intimate sexual behaviour on whether their partner were in agreement in their decision about their changing sexual behaviour.

*“…because we are done with childbirth, we decide to stop meeting as husband and wife… Then it is an agreement between us two for instance that we should withdraw”*(Awoke, M, 60–69).

By having mutual understanding, it affords the partners to jointly adopt and explore more ways to express their sexual behaviour as shown in the response of an older woman in remarriage:

*“…it takes mutual understanding to maintain a continuous sexual behaviour. …between the partner where they have accepted and learned how to adjust, how to compromise and how to accept [changes] together”*(Chioma, F, 80–89).

Those reporting in the contrary about being in agreement with their partner often mentioned issues such as lack of trust, personal concerns, and personal engagement as the more drive for their decision. This is demonstrated in the response of a male participant driver in a polygamous relationship:

*“…I made the decision alone… I am looking forward to living a longer life. Because …intercourse takes the blood of a man, which constitutes his strength… There is often behaviour from them [wives] that show they want it. Especially, the last wife; often she would be posing around but I always overlook it”*(Duruigbo, M, 70–79 ).

The driver’s response like some other men exemplified the characteristic patriarchal nature of the southeastern Nigerian traditional society where the men are revered and given total authority in the family, which showed to discourage mutual understanding.

The mention of the crucial influence of mutual understanding here is novel in this topic. There is only a mention of “mutual help” in the work of von Humboldt et al. (2021:7) without further interpretation on how it influences sexual behaviour. Meanwhile, mutual understanding showed an important influence on the other factors linked to older people’s sexual behaviour in this study, which gives it central importance on these factors (demography, motivations, partner factors, perception, and social influence). This finding could be of practical implication for use in therapeutic counselling sessions in helping the partners towards finding favourable adjustment in their sexual behaviour.

Major behaviour consistent with mutual understanding were love, trust, and self-control.

*“., through love and mutual understanding coupled with self-control, a woman naturally yields. I told you woman sexual behaviour is slow; you have to arouse her most times by being tender, getting playful, using polite words”* (Papa, M, 70–79).

*“Yes, one of the ways to maintain it, … is love, genuine love, agape love; you know it covers a multitude of sin… It makes the husband and wife be one. … Even the man is weak, a woman that loves you can come close play with you and help you get motivated and raise your urge…” (*Daberechi, M, 60–69*).*

Self-control was emphasized as a way to align mutually with each other’s sexual behaviour. Love was similarly identified in previous works to encourage later life heterosexual behaviour (Carvalheira et al., 2020; Agunbiade & Gilbert, 2020). In this study, as similarly reported by Tetley et al. (2018) women were most likely to talk about the importance of love and caring as the most required behaviour when the youthful fantasy and pleasure are diminished with ageing.

*“Love is a necessity because when there is love there is tolerance and more and this is mostly important in old age. People may pretend in the younger age but in older age, they cannot. As it were, older people live real life. The fantasies fade way, the realities surfaces”* (Chioma, F, 80–89).

Similarly, love was mentioned in the work of Lochlainn & Kenny (2013); Tetley et al. (2018) as a considerate behaviour that esteems each other. Additionally, trust was identified in previous studies to influence the depth of sexual relationship older people shared (Hinchliff & Gott, 2004; Štulhofer et al., 2020). Lastly, Freeman & Coast (2014) believes that self-control is a necessity as it allows partners to align mutually with each other’s sexual needs which agrees with the finding in this study. These behaviours applies in a varied way with different partners and they point to potential areas of intervention in partners who are having strains in their sexual behaviour.

### Mutual Understanding and Demographic Attributes

Mutual understanding was also reported to be linked to gender difference, family system, and religion of this study participants’. Mutual understanding helps in managing the difference in interest and motivations for sexual needs between the partners. Like, some of the interviewees mentioned that when a partner understands their gender differences, the man for instance could help the woman by deliberately motivating her sexual behaviour.

*“…through love and mutual understanding coupled with self-control, a woman naturally yields. …woman sexual behaviour is slow; you have to arouse her most times by being tender, getting playful, using polite words”*(Papa, M, 70–79).

Secondly, religion, especially Christianity exert obvious influences as many older people in this study identified belonging to one religious group and they are more drawn to act in conformity to religious norms and values in order to lead a good example. Christianity was reported to encourage mutual understanding as it teaches self-control and unity among partners as in the response of a Christian woman:

*“…togetherness of husband and wife brings happiness. Christianity talks about there should be no separation… Some men may still want to continue having sex; some women may not like it. But Christianity encourage understanding each other”*(Zubechi).

Unlike the report by Ginsberg et al. (2005), that religiosity is related to reduced sexual activity, Christianity showed in this study context to encourage sexual activities as it teaches monogamy, self-control, and unity, and that partners should not deny each other their sexual obligation. However, as was similarly reported by (Kolodziejczak et al., 2020), Christianity appeared to discourage older people’s option and choice for an extra-marital relationship and some alternative practices like masturbation, even when the older individuals want it, especially on the older women who often faces double expectation from being aged and being female. This is seen in the response of an older woman whose husband is bedridden:

*Sexual behaviour when you are married is a different ball game. You are stucked with your husband … and no extra-marital life”* (Oluchi, F, 80–90).

Christianity also appeared to be linked to low sexual interest, especially among men who engage in fasting or spiritual devotion confirming the finding of Ojo (2007). On the other hand, traditional religion as practiced by some southeastern Nigerian older people is reported to encourage sexual behaviour for the men than their women counterparts as it encourages polygamy, remarriage, and old age marriage only in the favour of the man, which is a form of sexism.

More so, the southeastern traditional religious setting originally defined social status based on the size of one’s family (Nduka & Ozioma, 2019). Meanwhile, like in the work of Agunbiade & Ayotunde (2012), family systems like polygamy showed among these study participants to likely discourage sexual behaviour, especially for the first wife who is ignored for the younger ones. As was noted in the response of a polygamous man:

*“…the last wife is the one I still see but very very rare and seldom”* (Duruigbo, M, 70–79).

### Mutual Understanding and Motives for Sexual Behaviour

Knowing how to motivate one’s partner encourages sexual behaviour. The motivating factors mentioned here include a woman appearing attractive and expressing her sexual needs. This corroborates with the finding of Delamater et al. (2019) in their study among heterosexual older women where they showed that physical attractiveness of the woman is an important motivator. The importance of physical attraction could be argued to be heightened in the recent decades due to the influence of media in representing beauty with the youthful age (Chepngeno-Langat & Hosegood, 2012). Meanwhile, men who understand how to make their wife happy or satisfies her in previous sexual experience is reported to motivate her easily. It is important that the woman attain orgasm in previous experiences (Townes et al., 2021). Also, the ability of the older people to understand the underlying cause of their partner’s non-reciprocation like in a case of past sexual abuse as demonstrated in the response by a polygamous man who married his first wife in the wife’s teenage age:

*“Some women have been abused even as a child…. rape, controversial marriage can affect the characteristics of sexual behaviour until later life. …if you are married to such partner if you have the understanding you will be able to help…”*(Nwanga, M, 70–79).

Many studies on sexual violence support the finding that the traumatic effect of sexual abuse in one’s earlier age could continue with physical and emotional consequences into the adult age (WHO, 2002). In forensic nursing perspective, for instance, it takes objective description and understanding of victims’ trauma and past sexual abuse to help them overcome it (Hazelwood & Burgess, 1995), which could be comparable to partners’ mutual understanding shown in this study.

More so, both partners require a mutual understanding of when they are in their good mood for sexual behaviour. For some participants, sexual behaviour is only possible in their leisure or free times. This is demonstrated in the response of a retired older woman:

*“Most times when we are happy, when we are lonely, and we try to be with each other”* (Oluchi, F, 80–89).

As in previous studies (McFarland, Uecker, & Regnerus, 2011; Fischer et al., 2021), the happiness of the older partners is reported as a key motivating factor but could be compromised by strains or crises in the home. In this study, the rising responsibilities of the older people were generally noted as a top priority than their sexual needs. This contradicts the finding of Delamater et al. (2019) who suggested that some older people become more sexually active in later years, as the empty nest and retirement provide them with more time and freedom to engage in sexual activity spontaneously. The difference in their finding could be specific to countries where a financially secure retirement is possible. Instead, marital happiness showed more motivating potency as in the work of Hincliff & Got (2004) who pointed out that happiness was linked to couples’ willingness to participate in leisure and activities that brought them closer together.

### Mutual Understanding and Partners Factors

Similarly, partner factors such as conflict and crisis, knowledge, and skills of partners about sexual practices, and presence of sexual dysfunction, menopause, and partner ill health were reported to influence older peoples’ sexual behaviour. For example, while most of the older people seem conservative in their approach to sexual relationships, few were willing to learn and explore more skills and approaches to their sexual behaviour as in the case of an older man who was exposed to sex education:

*“…education can make your brain and experience wider. …we learn by reading, …then we practice… just like menopause…. If you understand your spouse, you will know this and how it affects their sexual behaviour”* (Papa, M, 70–79).

Meanwhile, those that are more conservative believed that there is no way to encourage sexual behaviour because new behaviour is not learned in older age.

*“…it will have to be inculcated from the upbringing… There’s a saying in the Igbo language that they don’t learn left-handedness in old age… Even if one wants to do that, the person will appear funny”*(Chioma, F, 80–89).

More so, menopause, sexual dysfunction, and partner’s ill health often require adjustments in partners’ sexual behaviour to make up for the partner that have witnessed these changes. Where the partners are not able to adjust mutually, it discourages their sexual behaviour like in the response of a woman who reported her partner is frail:

*“[Laughs] I do not know how to answer it because, like now, is not that if my husband is ready and is not sick and can do it; I think I will be able too”* (Ngozika, F, 60–69).

### Mutual Understanding and Social Exposure

Societal influence such as the older person’s home training, and repressive education, gendered role, and its associated sexism in the older population are linked to their sexual behaviour depending on the extent of the partners’ mutual approach to it. For instance, across the transcripts, there was little mention of alternative forms of physical sexual activities like masturbation, oral sex, extra-marital practice, cuddling, and hugging. The interviewees hardly want to talk about them except on direct probes to verify if they elicit specific sexual behaviours. Only some interviewees who are less attached to socio-cultural norms consented that they often include hugging, kissing, cuddling as foreplay in their sexual activities:

*“…we have to start with the foreplay to prepare. Men are always aroused quicker than women are… Sexual interest can be inflamed/helped or aroused by the partner through caressing and others”*(Papa, M, 70–79).

Many other interviewees reported expressly that it is not common in the Igbo traditions and their upbringing to behave in those ways; it is unusual for parents to cuddle or hug their children, which then extends to the type of affection they seek later in life. This is seen in the responses of an older man and woman respectively:

***“****Kissing, hugging. Well those things is not in our culture. It is not in African culture [laughs]”*(Odogwu, M, 70–79).

*“…this age does not have it unless those who started it early. …the behaviour you acquired earlier can take time to change”*(Zubechi, F, 60–69).

Thus, for these participants, the nature of their sociocultural exposure could be the primary reason why alternative sexual behaviour is uncommon. And, it partly explains why behaviours may be compartmentalised. As a general pattern in this study, older people who are more conservative in approach are more likely to perceive sexual activities as non-beneficial for the ageing body. Especially, older men perceived sex to reduce longevity. They often argue that they had to abstain from penetrative sex to help prolong their life. This is similar to the finding of Vasconcelos et al. (2021); Gewirtz-Meydan & Ayalon (2020) that conservative sexual beliefs are a consequence of conservative upbringing. More so, the women showed to be more conservative and passive about sexual behaviour confirming the work of Ayalon et al. (2019).

Again, the southeastern older people aged 60 years and above are the cohorts of people that witnessed and survived the Nigerian civil war that deprived many of parental affection, as well as repressive, and punitive education.

*“They war taught us responsibility from a very early time in a very rough way…”* (Abara, M, 70–79).

The effect of this war and the traditional educational system they received, including isolated home training, could have had a conservative effect on their sexual behaviour. This supports a longitudinal studies in Europe that suggested that the Second World War had a conservative effect on the cohort of older people that experienced it (Kolodziejczak et al., 2020). This influence could persist in some until older age where it presents as low interests for sexual behaviours.

Yet, as is similarly reported by Hinchliff et al. (2020) about upbringing, who identified that the older people are ‘sandwiched’ between the restricted social standards of the past and the unrestrained present. There seems to be a weaker influence of education compared to that of upbringing and social exposure as the participants in this present study mostly confirmed that it is ridiculous to think of changes in behaviour in the later years. However, a few participants in this study who have re-learned through western education as with Odugwu or through much training on sexual topics as with Papa showed a more liberal approach in their sexual behaviour attributable to their continuous learning on sexual topics.

Furthermore, most of the women reported they are experiencing sexism. The patriarchal and religious society in southeastern Nigeria favours the sexual rights of the man over the woman. This is because it encourages polygamy, remarriage, and old age remarriage only in the favour of the man. This is demonstrated in the response of an older woman who remarried in later life:

*“…in African traditional religion…, the man is seen as a hero by his sexual capacity. An example is in the book ‘things fall apart’ by Chinua Achebe with the character ‘Okoro’. That book was written with the Igbos socio-cultural setting… where men are regarded depending on the number of children and wives they have due to… farming capacity…The original African tradition in the Igbo sense support sex once procreation is the essence otherwise… is term irresponsible for a man to engage in extra-marital affairs”(*Chioma).

Some women however, reported that they are not deprived of their right to make their sexual decisions.

*“The days I have reasons to say no, I do say no. though it does not often have him happy”*(Adaoma, F, 60–69).

Even though the influence of Christianity and modernization have reduced the practice of the polygamous family system, its associated patriarchal ideology, gendered expectations, and power relations about sexual rights and decision-making are still in existence, and links to sexism. The effect of sexism on older people’s sexual behaviour is captured by Heywood et al. (2019) who argued from Levy’s (2009) stereotype embodiment theory that these expectations become internalized by the older people in their quest to conform to the social norms.

### Study Limitations

The findings of this study have certain limitations that warrant caution. Firstly, the data collection was done by a single male researcher, which could have introduced some limitations in the data generated from the women as they were mostly more hesitant to answer some of the interviews questions. Thus, there could be gender bias in that there are more responses from men that shape the perspective in the data analysis. However, effort was made to overcome this limitation by sampling purposefully for more women and conducting a comprehensive and credible analysis that sought validation of the emerging themes from all categories of the sample (Mason, 2002). More so, those that accepted to grant the interview had good level of rapport created as they often take on a mentorship-type role in imparting some wisdom or life advice to the interviewer.

More so, combining the data from men and women in the study analysis is an important limitations since men and women in this study have had different socialisation and gendered experiences of sexuality and intimacy. Likewise, there is risk of homogenising the sexuality of older people as it includes participants of a wide age range (60–89 years) and both men and women in the analysis. However, efforts were made to avoid the general tendency to frame older people as all the same and their older age as their primary characteristic, especially as the finding here emphasized on the diversity of behaviour.

### Conclusions

Later life sexual behaviour is best described as diverse, older people in this study mostly adjusted to incorporate more companionship and loving care for each other. However, most time what forms of sexual behaviour constitute for these older partners are often link to the presence of dynamic interplay of influencing factors like their mutual understanding. Remarkably, these factors could be controlled, which signposts a potential premise and point of policy and practical intervention to promote healthy sexual behaviour in later life.

### Implication for Policy and Practice

Firstly, this study acknowledged the individuality and variability of sexual behaviour across older people such that in practice intervention to address the sexual needs of older partners requires a subjective assessment. For instance, a comprehensive assessment approach such as the ecological dynamic system theory is recommended (Jen, 2017).

Again, mutual understanding presents as a potent tool for counselling therapy for social workers and psychologists by actively collaborating with the partners to identify areas of mutual strength in their sexual behaviour where they can adapt and develop more in the face of challenges.

On sex education, this study finding indicates that programs that promote the values of older people’s sexual behaviour are warranted. Especially teaching on older people’s sexuality could help promote help-seeking from appropriate care agencies. Presently, most teaching and programs including sexual education seem to be pointing to the adolescents, youths and for procreation or family planning needs, with little or no attention to the older people, awareness for sexual education for the older people is hereby recommended for policy consideration.

Finally, there is a policy need to reverse social attitudes and expectations about older peoples’ sexual behaviour by creating educative and awareness programs focused on enlightening the public on the benefit of sexual health rights for healthy ageing. Besides, given the World Health Organization acknowledgment of the rights of all people to be sexual regardless of age (WHO, 2006), there is a heightened need to review the Commonwealth Sex Discrimination Act of 1984 to advocate and ensure the sexual health of the older people as a rights issue. The Commonwealth Sex Discrimination Act of 1984, which is accepted generally by her member nation is presently focused on “supporting women’s rights, eradicating sex-based discrimination, and discrimination regarding sexual rights in the areas of public life“( Ashford, 2001 cited in Chepngeno-Langat & Hosegood, 2017:54). There is yet little attention given to the sexual behaviour of older people, which evidence from this study suggests for its advocacy.

### Recommendation for Future Studies

In this study, some of the men argued that they looked better when they abstained from sexual intercourse, a further study to verify the beneficial effect of sexual intercourse among older men in the sub-Saharan African is warranted. Similarly, an interesting linking of sexuality with strength was highlighted across several of the extracts in several themes, which needs future research to examine this more carefully. As well, couples could be interviewed together as sexual behaviour occurs within partners and could generate elaborated responses together. Future study is also needed around polygamy and paying sexual attention to different wives. Finally, due to the importance of study context in the understanding of later life sexual behaviour, more study on this topic is recommended in other sociocultural settings.

## Data Availability

Not applicable.
